# Anomalous intralayer growth of epitaxial Si on Ag(111)

**DOI:** 10.1038/s41598-024-52348-1

**Published:** 2024-01-29

**Authors:** Kejian Wang, Geoffroy Prévot, Jean-Noël Aqua

**Affiliations:** Sorbonne Université, Centre National de la Recherche Scientifique, Institut des NanoSciences de Paris, INSP, 4, place Jussieu, 75005 Paris, France

**Keywords:** Two-dimensional materials, Thermodynamics

## Abstract

The epitaxial growth of silicene has been the subject of many investigations, controversies and non-classical results. In particular, the initially promising deposition of Si on a metallic substrate such as Ag(111) has revealed unexpected growth modes where Si is inserted at the beginning of the growth in the first atomic plane of the substrate. In order to rationalize this anomalous growth mode, we develop an out-of-equilibrium description of a lattice-based epitaxial growth model, which growth dynamics are analyzed via kinetic Monte-Carlo simulations. This model incorporates several effects revealed by the experiments such as the intermixing between Si and Ag, and surface effects. It is parametrized thanks to an approach in which we show that relatively precise estimates of energy barriers can be deduced by meticulous analysis of atomic microscopy images. This analysis enables us to reproduce both qualitatively and quantitatively the anomalous growth patterns of Si on Ag(111). We show that the dynamics results in two modes, a classical sub-monolayer growth mode at low temperature, and an inserted growth mode at higher temperatures, where the deposited Si atoms insert in the first layer of the substrate by replacing Ag atoms. Furthermore, we reproduce the non-standard $\Lambda$ shape of the experimental plot of the island density as a function of temperature, with a shift in island density variation during the transition between the submonoloyer and inserted growth modes.

## Introduction

Since the isolation of graphene flakes thanks to exfoliation, 2D materials have aroused an ever-growing interest as different materials can form 2D layers (e.g. carbon, silicon, transition metal dichalcogenides...)^1–6^. They are expected to potentially surpass all previous technologies thanks to their outstanding electronic properties, and their integration in devices has attracted numerous studies. Graphene is probably the most studied material worldwide, due to its unique properties related to the Dirac-cone-shaped energy bands at the Fermi level and high carrier mobility. However, despite significant efforts, there has been no reproducible method to open up its bandgap while preserving high carrier mobility. 2D Materials (2DM) based on group IV elements such as Si (silicene) and Ge (germanene) are promising alternatives^7^. They are predicted to be Dirac materials in which electrons behave as relativistic massless particles, and, more importantly, are better suited than graphene in the race for ultimate thickness scaling of nanoelectronic devices. Obtaining 2DM by the common exfoliation technique has intrinsic limitations, notably on the size and quality of the 2D crystals thus obtained, and is not appropriate for silicene or germanene that do not exist in nature in allotropes of Si or Ge. The remaining alternative is the production of 2DM by epitaxy that is a technological lock for any potential industrial application of these systems. A huge experimental effort has been dedicated to the epitaxial growth of silicene but is still ongoing as no reliable, high-quality and large-scale method is available. Some results are controversial, others are not conclusive. Epitaxial growth of silicene was first reported on metallic substrates. The deposition of Si on Ir(111) led to a layer of Si atoms strongly bonded and hybridized with substrate atoms^8,9^. The deposition on Ru(0001) led again to a 2D film but with no Dirac cones due to charge transfert with the substrate atoms^10^. The deposition on Ag(111) and (110) led to a long-standing controversy as linearly dispersing bands at the Fermi level were first reported as being Dirac cones, but turned out to result from the strong coupling between Si and Ag atoms^11–20^. In any case, the understanding and control of the epitaxial growth of 2D materials is largely insufficient, and today’s progress is limited by the lack of wafer-scale uniform growth that requires further investigation of dynamical mechanisms.

To progress in this direction, we develop numerical simulations of the epitaxial growth by considering a concrete example that has given rise to many experiments, the growth of 2D silicium on Ag(111). Experiments revealed the importance of the film/substrate interactions, but also exotic growth modes in some conditions, and especially in the early stages of growth in the submonolayer regime. Even though not miscible in bulk, Si and Ag were indeed found to significantly intermix at the film/substrate interface and even to lead to Si islands inserted in the substrate in some temperature range. Only a few theoretical works have been devoted to the study of the epitaxial growth of 2DM,^21,22^, and even less on silicene or germanene. First principles-calculations have revealed the stability of structures^17^, but the dynamical description of these systems is sparse due to major difficulties. The challenge is to simulate out-of-equilibrium systems of sufficient size (typically of the order of a hundred nanometers) yet incorporating atomic details, over sufficiently long times (typically of the order of a minute or more) yet describing atomic events (diffusion, incorporation, exchange...). These space and time scales are unreachable with usual ab-initio or molecular dynamics tools, but kinetic Monte-Carlo simulations of lattice models allow us to do the splits between these scales thanks to a probabilistic description of microscopic transitions. We thence develop here both a new modelization of the growth dynamics and extensive simulations in order to rationalize the anomalous growth modes of Si on Ag(111). We show that both intermixing and layer-dependent energetics can rationalize both the growth modes and its statistical properties.

## Methodology and results

### Experimental results

**Figure 1:**
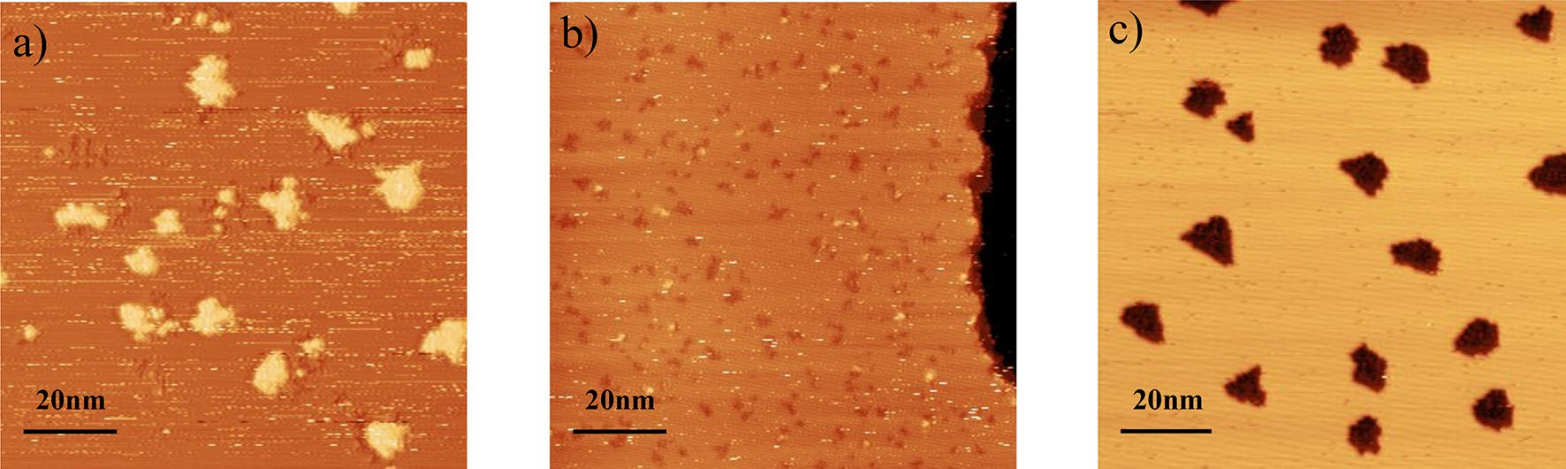
STM investigation of the growth of 0.3 ML of Si on Ag(111) at different temperatures $T \! = \!198$ K (**a**), 300 K (**b**) and 440 K (**c**). In (**a**), the darker zone correspond to the substrate, and lighter zones, to the Si island growing on top of the substrate. In (**b**) and (**c**), the darker zones stand for inserted Si atoms, while lighter zones stand for the Ag substrate.

We briefly recall the highlights of the anomalous growth of Si on Ag(111) described in Ref.^17^. Although Si and Ag are not miscible, the deposition of Si on Ag(111) revealed two temperature-dependent growth modes where the deposited Si atoms can be inserted into the Ag substrate, see Fig. 1. At low temperatures ($T \! = \!200$ K), a classical growth occurs where two-dimensional islands nucleate on the substrate surface and then grow during further deposition, as in the homoepitaxial sub-monolayer epitaxial growth. On the other hand, for higher temperatures ($T \ge 300$ K), Si atoms insert themselves into the first layer of the substrate and expel the Ag atoms which diffuse to accumulate at the step edges. For increasing Si deposition, this nucleation phase is followed by a growth phase where the islands expand in the first layer of the substrate, by continuously expelling Ag atoms. This island growth regime ends with a coalescence regime for which the island density decreases up to the completion of a complete two-dimensional Si layer on the substrate.

**Figure 2:**
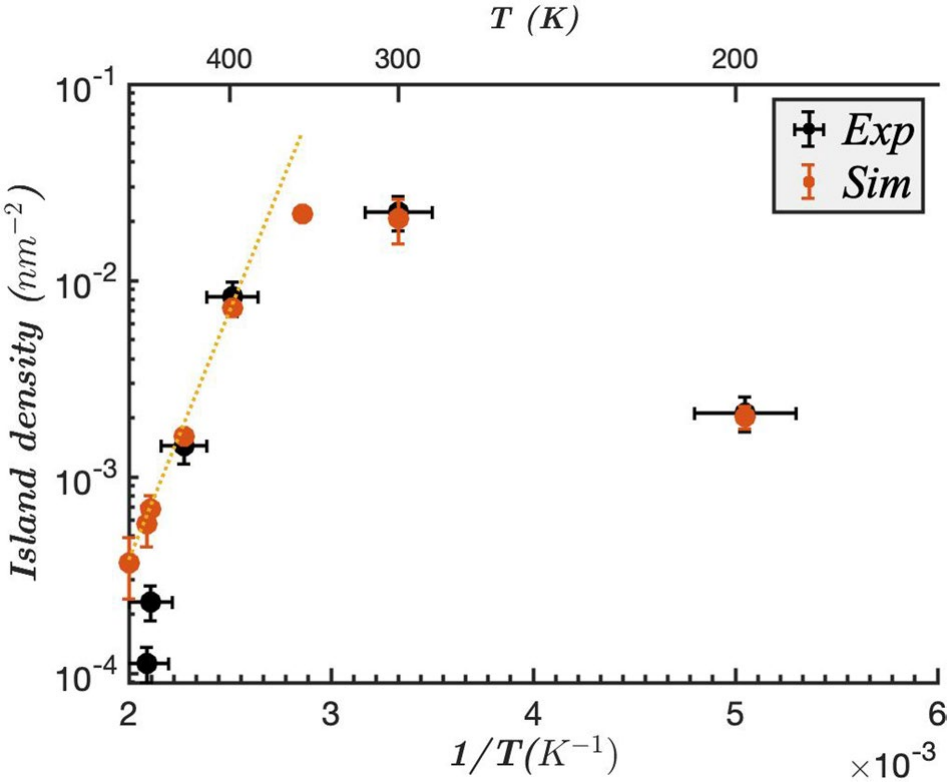
Island density as a function of temperature for a deposit of 0.1 ML of Si on Ag(111), for a given deposition flux of 0.1 ML/h, for $T\! = \!198, 300, 398, 440, 474, 478$ K (experimental values of Ref.^17^). The black points correspond to experimental values, while the orange points for Kinetic Monte-Carlo simulations (with the two extra temperatures 350, 460 and 500 K) with the optimized parameter set described in the following.

The variation of the island density in the island growth regime with temperature is also abnormal, see Fig. 2. The density of islands strongly increases between $T\! = \!200$ K and 300 K, by a factor of 10. Then, the island density decreases with temperature above 300 K, in the abnormal growth regime where the islands are inserted in the first layer of the substrate. This latter decrease is usual, and corresponds to the fact that with increasing temperature, the deposited adadoms can further diffuse before being captured by an already nucleated island, giving rise to larger and less dense islands (for a given amount of deposited material). Yet, the strong increase of the density between 200 and 300 K is abnormal, even if already observed in some heteroepitaxy on metallic substates, e.g. Pb/Cu(110) and Ni/Ag(111)^23,24^. It corresponds to the effects of intermixing and insertion of atoms in the substrate for temperatures high enough to pass the insertion barriers. As a whole, the unsual $\Lambda$ shape variation of the Arrhenius plot of the island density was attributed to the competition between the insertion of isolated Si atoms and the mobility of these atoms.

### Modelization

#### Kinetic model

**Figure 3:**
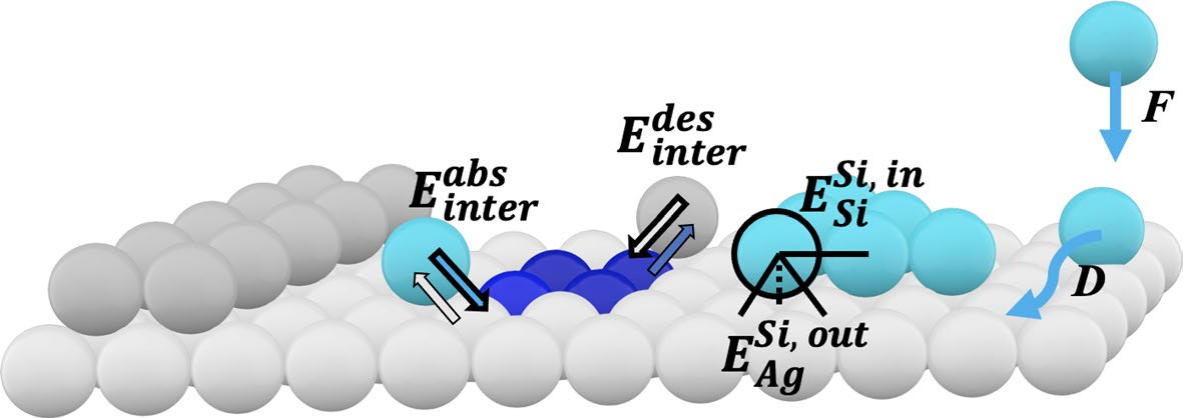
Geometry of the system: Si atoms (in blue) are deposited with a constant flux *F* onto a Ag(111) substrate (light grey atoms) with a step edge (dark grey). Deposited atoms first attach as adatoms (light blue), but may undergo intermixing and thence incorporate in the substrate (dark blue) with an energy barrier $E_{inter}^{abs}$. An inserted Si adatom (dark blue) may also exchange with a Ag adatom (light grey) with an energy barrier $E_{inter}^{des}$, and become a Si adatom. The contribution to the energy barrier of a given atom of species $\sigma$ due to its in or out-of-plane neighboring atoms of species $\tau$ is $E^{\sigma , in/out}_{\tau }$ (by convention, out-of-plane links are always with the $\sigma$ atom above the $\tau$ atom). The Si and Ag lattices are supposed pseudo-morphic and follow the substrate fcc (111) lattice.

We consider a lattice model suitable for performing kinetic Monte-Carlo simulations of epitaxial growth^25–35^. We consider a face-centered cubic lattice bonded by a (111) surface and assume a pseudomorphic growth, see Fig. 3. Every site can be either empty, or populated either by a Si or Ag atom. We assume that every atom is bonded only to its nearest neighbors. Yet, the atom binding energies generally depend on the atomic configuration, and may be layer-dependent and species-dependent, thus introducing surface and composition effects.

The dynamics is defined on the basis of a Markovian random process characterized by characteristic frequencies. We consider as elementary processes diffusion, attachment, detachment, intermixing and the atomic deposition characterized by the flux *F* in monolayer (ML)/s, see Fig. 3. Thermal fluctuations are embedded in the vibrational frequency $\nu _0 = k_{\scriptscriptstyle {B}}T / h$. Except for the atomic deposition, the elementary processes are characterized by a rate $\alpha \, \nu _0 \exp \Delta E$, where $\Delta E$ is the energy barrier to be overcome for the process to occur^26,29,36–39^, and $\alpha$ is a prefactor that may depend on the species of the atom under consideration. We consider the energy barriers corresponding to the bond breaking of the nearest-neighbor interactions, thus satisfying detailed balance. Interactions may be (1) anisotropic depending whether it is in-plane or out-of-plane, (2) species-dependent according to the Si or Ag atoms involved in the bond and (3) layer-dependent according to the height of the atom with respect to the surface. Hence, in the general case, we assume that the elementary energy barrier for both diffusion, attachment and detachment of a tested atom of species $\sigma =\{$Si, Ag$\}$ reads

1 $$\begin{aligned} \Delta E^{\sigma } = \sum _{\tau =\{{\tiny {Si, Ag}}\}} \left[ n^{in}_{\tau } E^{\sigma , in}_{\tau }(h) \right. \left. + n^{out}_{\tau ,\uparrow } E^{\sigma , out}_{\tau }(h+1) + n^{out}_{\tau ,\downarrow } E^{\sigma , out}_{\tau }(h) \right] \,. \end{aligned}$$

The sum runs over the species of the nearest-neighbors, either in-plane (*in*) or out-of-plane (*out*), while $n^{in}_{\tau }$ is the number of in-plane nearest-neighbors of species $\tau$, $n^{out}_{\tau ,\uparrow }$ (resp. $n^{out}_{\tau ,\downarrow }$) is the number of out-plane nearest-neighbors of species $\tau$ above (resp. below) the tested atom. To avoid ambiguity, the barrier $E^{\sigma , in}_{\tau }$ always concerns a bond pointing downwards between an atom of species $\sigma$ lying on top of an atom of species $\tau$. In addition, we also introduce surface (or wetting) effects by considering that the energy barrier per bond $E^{\sigma , in/out}_{\tau }$ as well as the prefactors $\alpha ^{\sigma }$ may depend on the height of the tested atom. For simplification, we account only for two sets of energy barriers, one for $h\le 0$ that characterizes atoms in the substrate (either Ag atoms or possibly inserted Si atoms), and one for $h \ge 1$ that characterizes atoms on top of the substrate. This restriction is not restrictive, since we will only be considering sub-monolayer growth, where in practice the maximum height is essentially $h \! = \!1$. As regards intermixing between Si and Ag atoms, we consider that it is characterized by an energy barrier $E_{inter}^{abs}$ for the absorption of a Si atom on top of the surface with a Ag atom beneath, and by $E_{inter}^{des}$ for the possible desorption and interchange of an inserted Si atom below a Ag adatom on the surface. Considering all the above mentioned possible processes for every atom, including deposition, we can build a ladder of rates and randomly select the elementary moves. We use in the following the rejection-free continuous-time Bortz-Kalos-Lebowitz algorithm^40^.

#### Parametrization

In order to parameterize this model, we have developed a new approach that allows us to estimate energy barriers with relative precision. Parameterization of this model is complex since it is a priori characterized by 11 parameters for each height *h*: 2 amplitude coefficients $\alpha$ for Si and Ag, 3 in-plane barriers (Si–Si, Ag–Ag, and Si–Ag or Ag–Si which are equal), 4 downward facing out-of-plane barriers (Si on Si, Si on Ag, Ag on Si, Ag on Ag), and 2 inter-mixing barriers ($E_{inter}^{abs/des}$). In addition, most of these coefficients have an impact on the morphological and statistical results of the model. In these conditions, reproducing experimental results qualitatively and quantitatively is a tour-de-force that we achieve thanks to the meticulous inspection of the morphologies revealed by atomic microscopy. Indeed, comparison of these images with the model results lead to relatively precise estimates of energy barriers (to within 0.01eV typically) which forms a set of values reflecting the system.

We proceeded by intuiting a starting parameters set through literature (both experimental estimates and ab-initio calculations) and comparison with the morphologies of Si deposits on Ag(111), and then, thanks to a back-and-forth approach, by fine-tuning the set of parameters to reproduce both qualitatively and quantitatively the experimental results. The first set of parameters allows the main qualitative aspects of the system to be reproduced by analyzing various morphological details of the system, see Supplementary Materials: convex vs dendritic vs facetted shapes of 2D islands, decoration of the step edges, 2D vs 3D shapes, voids in the first layer, intermixing in the islands, etc. If this first set of parameters allows to obtain the qualitative characteristics for the growth of Si on Ag(111) (i.e.  islands shapes, step edge decoration, island insertion in the first layer only for $T\ge 300$ K etc), it does not allow us to obtain the quantitative predictions of island densities as a function of temperature. In a second step, we therefore refined the values of the different energy barriers, in order to (1) keep all the qualitative features and (2) reproduce the variation of the island density with temperature. We were able to start from the low temperature $T\! = \!200$ K where the intermixing is negligible, in order to essentially refine the parameter $\alpha$ as well as the energy $E^{Si,in}_{Si} (h \ge 1)$. In particular, we have increased the energy barriers $E^{Ag,in}_{Si} (h\le 0)$ and $E^{Si,in}_{Si}$ in order to stabilize the Si atom insertions that form in the first layer of the substrate for $T \ge 300$ K, and slightly refined $E^{Si,out}_{Ag}$. Eventually, we obtained the optimized parameter set given in the Table 1. It is worth noting that these energy barriers are the crucial parameters of the out-of-equilibrium statistical physics on which this model is based, and of thermodynamics analysis^26,36–38,41^. Their values are here deduced from experiment rather than induced by atomistic approaches that could, for example, be obtained by calculations coming from the density functional approximation. Ab-initio results available in the literature were incorporated as initial constraints in the search for a set of parameters consistent with experiments. Energy barrier calculations require the exploration of all diffusion pathways, not just local energy minima, and their reliability requires careful analysis^42–44^. While we do not have the full calculation of all the coefficients of the model, we argue that the process of deducing parameters from the out-of-equilibrium analysis is compulsory in order to find a set of barriers that rationalize experiments (Supplementary Information).

**Table 1:** Optimized parameter set that reproduces both the qualitative morphological features of the Si/Ag(111) epitaxy (i.e.  islands shapes, step edge decoration, island insertion in the first layer only for $T\ge 300$ K etc), and the quantitative evolution of the island density as a function of temperature.

Energy barriers (eV)	$\quad h \le 0 \quad$	$\quad h \ge 1 \quad$
$E^{Ag, in}_{Ag}$	0.35	0.35
$E^{Ag, in}_{Si}$	0.065	0.035
$E^{Si, in}_{Si}$	0.38	0.088
$E^{Ag, out}_{Ag}$	0.03	0.03
$E^{Ag, out}_{Si}$	0.03	0.03
$E^{Si, out}_{Ag}$	0.122	0.10
$E^{Si, out}_{Si}$	0.12	0.12
$E^{abs}_{inter}$	1	0.50
$E^{des}_{inter}$	1	0.60

### Discussion

**Figure 4:**
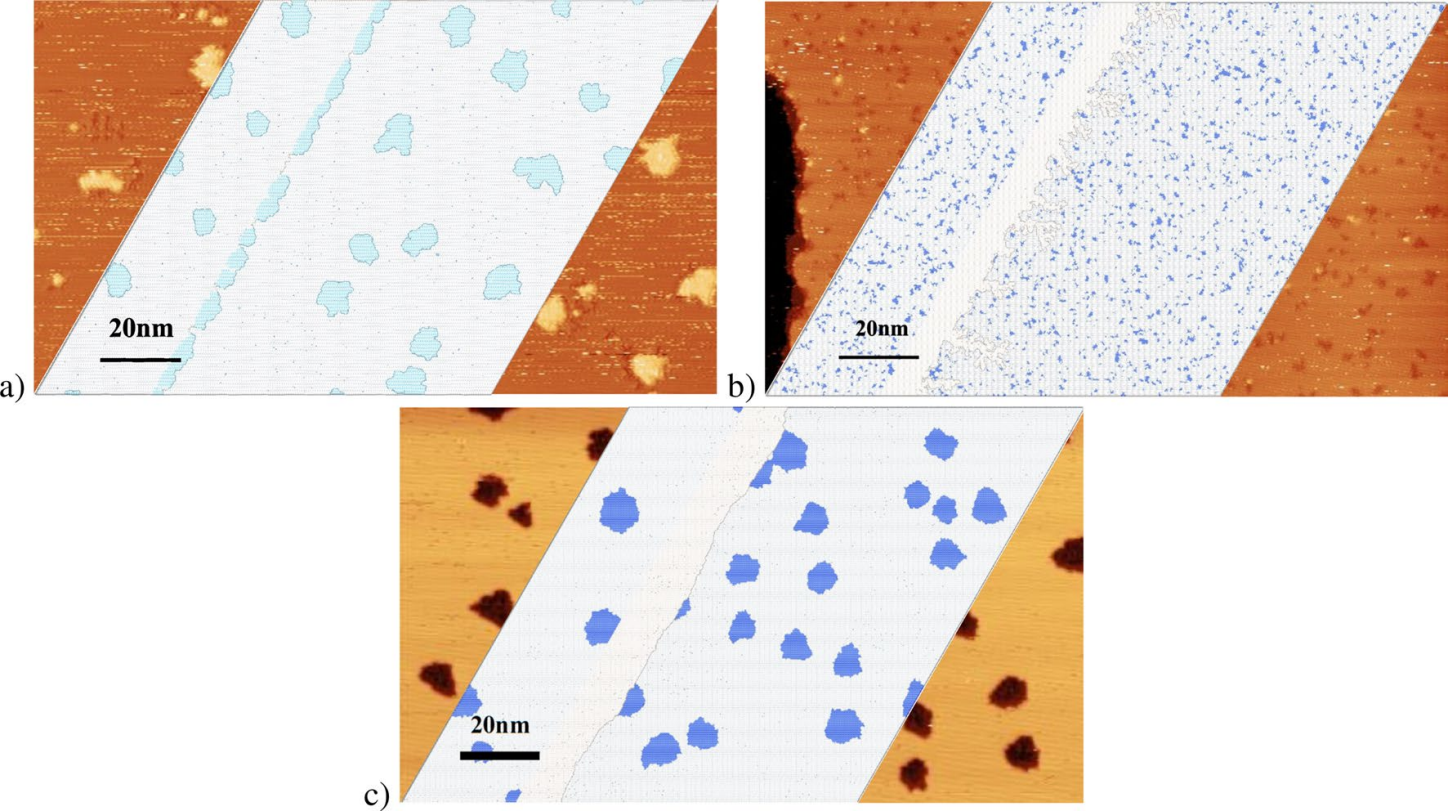
Simulations of the epitaxy of 0.1 ML Si on Ag(111) for $T\! = \!198$  K (**a**), 300 K (**b**) and 440 K (**c**). The deposition flux is 0.1 ML/h. The atoms in grey are the Ag atoms, those in brown/cyan, the Si atoms on the substrate and finally those in dark blue, the Si atoms inserted in the first layer of the substrate. The simulation box is $400\!\times \!400$ in lattice units on a honeycomb lattice. Colored insets show the experimental morphologies found at these conditions in^17^ scaled up to the same size.

**Figure 5:**
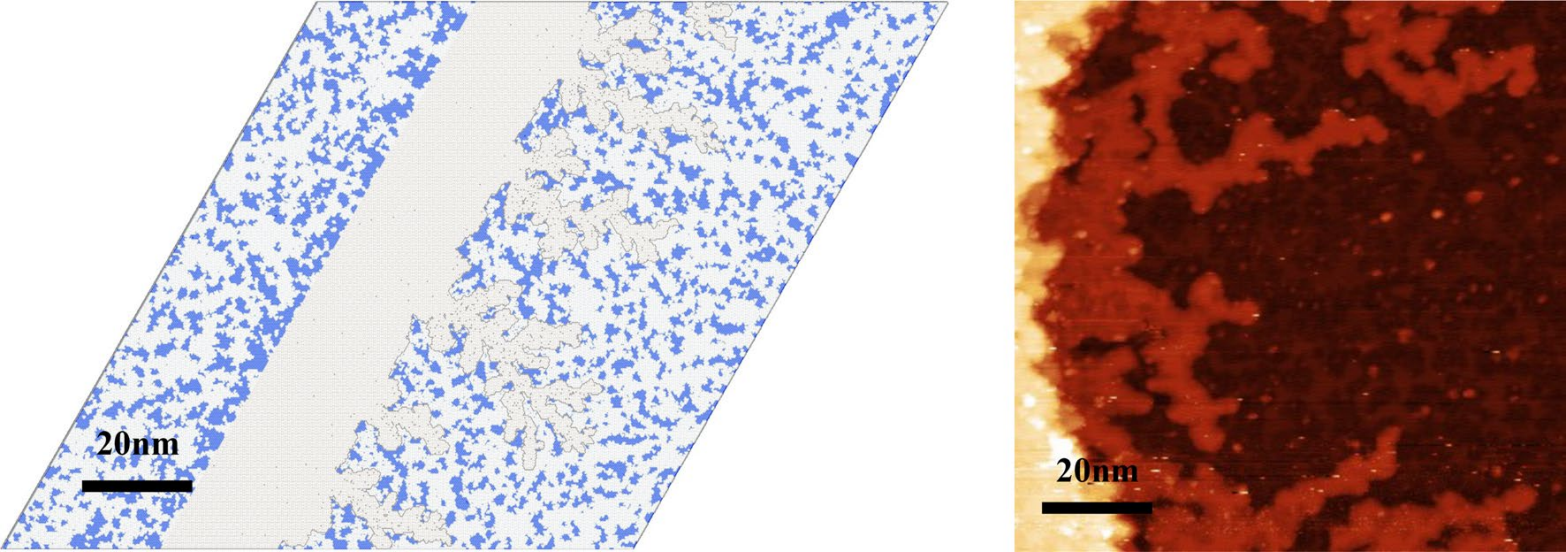
Vicinity of a step edge (left) for the simulation of the Si/Ag(111) epitaxy at $T\! = \!300$ K and conditions of Fig. 4 and (right) as found in the experiments in^17^ at this temperature. Ag atoms expelled from the first layer of the substrate by the insertion of Si atoms into the first layer of the substrate, accumulate at the walking edge, and form dendrites at this temperature. The Si islands are inserted in the first layer of the substrate. These two characteristics are consistent with the experimental results. The experimental inset represents a large scale scan where light blue dendritic shapes stand for Ag atoms accumulating at a step edge.

We plot in Fig. 4 the morphologies resulting from the kinetic Monte-Carlo simulations of the model exposed above with the optimized parameter set. It reproduces qualitatively well the features of the experimental anomalous growth of Si on Ag(111) with: (1) two-dimensional islands of Si that sit on the substrate at low temperature, $T\! = \!200$ K, with compact and irregular shapes, and a continuous decoration of the step edges; (2) islands that grow in the first layer of the substrate for $T\! = \!300$ K and above, with a density much higher than that of the islands at low temperature; (3) a density of inserted islands that decreases with temperature; (4) an accumulation of Ag at the step edges above $T\! = \!300$ K, see Fig. 5, which forms dendrites at 300 K and a continuous stripe at 400 K. In addition, the island densities (either sitting on the substrate for $T\! = \!200$ K, or inserted for $T\!\ge \! 300$ K) are shown in Fig. 2 (the results are obtained on a $400\!\times \!400$ system and averaged over 10 realizations of the dynamics with different random number seeds; Similarly to the experiments, we only compatibilized islands larger than $1nm\!\times \!1nm$). They reproduce quantitatively well the experimental densities with a 10-fold increase in island density between 200 and 300 K, and then a decrease in the inserted island densities at higher temperature as the diffusion length increases and coarsening occurs. The agreement is almost perfect for *T* between 200 and 440 K, and remains approximate at very high temperature for $T\!\ge \!474$ K. For these very high temperatures, the density of islands is small and finite size effects are introduced in the simulations. Moreover, the measure of the high temperature introduces a higher uncertainty on the results, in particular with regard to its dependence on step density. Yet, apart from these very high temperatures, the quantitative agreement between the experiments and the model can be considered as very good and validates the parameters incorporated in the model.

These results validate the relevance of the effects incorporated in the model used. We looked for a minimal model describing the basic and unavoidable mechanisms of epitaxial growth (deposition, diffusion, attachment-detachment), while adding the intermixing observed experimentally, and simple surface effects (with two configurations $h\! \le \! 0$) and $h\! \ge \! 1$). It is noticeable that surface effects (or wetting effects) were found to be unavoidable to reproduce qualitatively the growth modes. Altogether, intermixing and wetting appear to be the keys to the abnormal growth patterns of Si on Ag(111). The energy barriers described in the Table 1 thus reproduce these results well and are constrained by this statistical analysis and the experimental results. The number of parameters leaves some latitude on the choice of precise values of these parameters, which must therefore be understood with some uncertainty. Nevertheless, as described in the parameterization of the problem, even small variations of 0.01 eV for some features (e.g. $E^{Ag,in}_{Si}$) or 0.05 eV for others (e.g. $E^{Ag,in}_{Ag}$), can lead to results qualitatively incompatible with the experiments, thus limiting the uncertainties. Finally, it is notable, see Fig. 2, that the simulation results show a linear dependence for the Arrhenius plot of the density as a function of temperature for high temperature ($T \ge 400$ K), with a typical activation energy of $0.53 \pm 0.08$ eV. This result cannot be compared with the experimental results because of the smaller number of points measured.

## Conclusion

The growth of Si on Ag(111) revealed experimentally an anomalous growth pattern where islands of Si insert into the first layer of the substrate at high-enough temperatures even though Si and Ag are not miscible in the bulk. To rationalize this new growth mode, we derived a kinetic lattice model describing the main effects of epitaxy (deposition, diffusion, attachment-detachment) while incorporating intermixing and wetting effects. The growth dynamics of the model was computed by kinetic Monte-Carlo simulations. The parametrization of the model was derived using an approach combining meticulous analysis of atomic microscopy images and statistical properties as a function of temperature, and allowed to reproduce both qualitatively and quantitatively the experimental results, thus validating the main ingredients of the model at the origin of the growth mode. This first study allows us to lay the foundations for the study of the growth of thicker deposits which have revealed possible islands buried under the substrate, as well as Si or Ge on other metals such as Ag(100) or Au(111) which are the subject of current experimental investigations.

### Supplementary Information

Supplementary Information.

## Data Availability

The datasets used and/or analyzed during the current study are available from the corresponding author upon request.
