# Supplementary material for: Anomalous intralayer growth of epitaxial Si on Ag(111)
Source: Sci Rep. 2024 Jan 29;14:2401. doi: 10.1038/s41598-024-52348-1 (PMC10825137; doi:10.1038/s41598-024-52348-1)
Supplement: Supplementary file 1 — Supplementary Information. [file 41598_2024_52348_MOESM1_ESM.pdf]

Supplementary Materials

In this appendix, we justify in detail the choices we have made to parameterize the model. To begin with, some data are available in the literature. The self-diffusion of Ag adatoms on Ag(111) was precisely studied and characterized by a low diffusion barrier per bond $E_{Ag}^{Ag,out}(h=1)=0.03\,\mathrm{eV}$ and a frequency coefficient $\alpha \simeq 0.05$[1]. Without further information, we consider this value for α for all atomic processes in the following. Similarly, the intermixing and attachment energies of Si on Ag could be deduced from the above mentioned experiments revealing the atypical character of this epitaxy[2]. The typical values that could be deduced were $E_{Ag}^{Si,out}(h=1)=0.09\,\mathrm{eV}$, while $E_{inter}^{abs}=0.43\,\mathrm{eV}$ and $E_{inter}^{des}=0.54\,\mathrm{eV}$. We remind that, by construction, this value of the $E_{Ag}^{Si,out}$ binding energy is only the contribution to the diffusion barrier of a single atomic bond (it being understood that each diffusing adatom has three such bonds).

In addition, other barriers could be deduced from various morphological observations that are notably dependent on the energy barriers, and that can help getting fairly accurate estimates of these barriers. We can thus observe, cf Fig. 3 in Ref.[2], that the Ag adatoms accumulate on the step edges and crystallize in dendritic forms. By simulating the deposition of silver atoms only, at room temperature and with the experimental flux of 0.1 ML/h, we effectively obtain the accumulation of Ag atoms at the step edges, with the strip-like equilibrium shape along the step for low values of $E_{Ag}^{Ag,in}$ (below 0.25 eV), dendritic shapes when $E_{Ag}^{Ag,in} \simeq 0.35\,\mathrm{eV}$, and fine fractal shapes for $E_{Ag}^{Ag,in} \gtrsim 0.40\,\mathrm{eV}$, cf Fig. 1. In the following, we consider the intermediate value $E_{Ag}^{Ag,in} \simeq 0.35\,\mathrm{eV}$ as the starting value for the parameter search.

Other energies barriers can be deduced from morphological observations at $T=200\,\mathrm{K}$ where intermixing is not at work, thus simplifying the analysis. It is the case for $E_{Si}^{Ag,in}=E_{Ag}^{Si,in}$ that we can a priori estimate low, given the weak affinity between Si and Ag. But we can also observe experimentally that during the deposition of Si at $T=200\,\mathrm{K}$, that the islands decorate the step edges in a continuous way, see upper part of Fig. 1a in Ref.[2]. When simulating the deposition at that temperature and for the experimental flux of 0.1 ML/h, cf Fig. 2, we find a poorly decorated step edge for very low energies, continuously decorated step edges between 0.03 and 0.04 eV, and intermixing in the islands beyond 0.05 eV which is not observed experimentally. We therefore retain the intermediate value 0.035 eV as a starting value for this energy.

Another example concerns the energy barrier $E_{Si}^{Si,in}(h=1)$ between Si atoms on the Ag surface that can be deduced from islands shapes. One can notice that the crystallized Si islands still at $T=200\,\mathrm{K}$ have compact and irregular shapes, neither facetted like those close to equilibrium, nor fractal like those strongly out of equilibrium, cf Fig. 1a in Ref.[2]. The simulation of the deposition of 0.3 ML of Si on Ag for $T=200\,\mathrm{K}$ and the experimental flux, cf Fig. 3, naturally gives facetted shapes close to equilibrium for low values of this energy (since thermal fluctuations allow the atoms to detach themselves, to explore the phase space and to find *in fine* the equilibrium shapes). Conversely, for high values of this energy, we find fractal shapes corresponding to an almost irreversible attachment that freezes the atoms as soon as they are first crystallized and leads to fractal shapes in a classical way. Finally, for intermediate values, we find compact and irregular shapes similar to the experimental ones. We therefore start from the typical value of 0.11 eV for this energy. Finally, it can be observed at $T=200\,\mathrm{K}$, that the deposition of 0.8 ML of Si results in islands that are already starting to grow their second layer. The simulations reproduce this feature when $E_{Si}^{Si,out}(h=1)$ is larger than 0.12 eV, see Fig. 4, and we start with this value in the starting set of parameters.

All the values indicated above constitute a first set of parameters that we tested by neglecting the surface effects, i.e. by considering that the energies do not depend on h. It leads nevertheless to an undesired configuration: by simulating the system at 300 K, we obtain Si islands which do insert in the first layer of the substrate in accordance with the experiments, but which do it only in the vicinity of the step-edges where there is an accumulation of Ag adatoms expelled from the substrate, see Fig. 5. This undesired effect is due to the low binding energy $E_{Ag}^{Si,in}$ (which was deduced from the shape of the step edge decoration). This low energy does not allow the Si atoms once inserted to remain long enough in the substrate: they insert themselves then leave almost immediately, leaving many voids in the first layer of the substrate. Yet, the bonding energy of Si and Ag atoms on the surface of the substrate is not necessarily equal to that of these same atoms in the substrate. We are thus naturally led to incorporate different energies according to the height, i.e. to consider surface effects. We simplify them by considering a set of barriers for the adatoms that diffuse above the substrate surface, i.e. for $h \geq 1$ (h being counted relative to the substrate taking into account a possible step), and another set of energy barriers for the atoms embedded in the substrate, i.e. for $h \leq 0$. Yet, as a first approximation, we can neglect these effects for Ag atoms that are in homoepitaxy and thus consider $E_{Ag}^{Ag,in/out}$ energies as independent of h, simplifying the parameter search. Similarly, we neglect the variation with h of $E_{Si}^{Si,out}$, as we are looking for configurations where Si atoms essentially insert themselves only in the first plane of the substrate at $h=0$, making the energy $E_{Si}^{Si,out}(h \leq 0)$ never required. Similarly, intermixing occurs only between the surface and the first layer of the substrate. The intermixing phenomena between the internal layers of the substrate can thus be neglected and we consider large energy barriers $E_{inter}^{abs/des}(h \leq 0)$ in order, de facto, to block these processes. As regards these energies for $h=1$, we consider $E_{inter}^{abs}=0.50\,\mathrm{eV}$, slightly higher than the value 0.43 eV of Ref.[2]. The latter value was estimated assuming an irreversible growth which is clearly not the case regarding the experimental island shape, and thence represents a lower bound for this energy barrier. In addition,

the value 0.50 eV avoids the simulations to produce too frequent absorption/desorption events. Finally, we consider E_{inter}^{des} that is still 0.10 eV higher than E_{inter}^{abs} as in[2]. Finally, we note that the values of energy barriers for intermixing in the deep substrate layers $E_{inter}^{abs}(h \leq 0)$ and $E_{inter}^{des}(h \leq 0)$ are only indicative. Indeed, their values here is just high enough so that they ensure that Si atoms inserted into the substrate do not penetrate deeper into the lower levels at the temperatures considered; without further experimental data, statistical analysis can only give this minimum barrier value.

The final set of parameters given in Table 1 is eventually obtained by quantitative comparison with the density vs. temperature curve, as explained in the main body of the article. It is interesting to compare our results with some results found by first-principle calculations. Ratsch et al. found in[3] that the energy barrier is 0.081 eV for the diffusion of Ag adatoms on Ag(111) that compares favorably with the diffusion barrier in our model $3 \times E_{Ag}^{Ag,out}(h=1) = 0.09$ eV, Table 1, while the experimental value is 0.097 eV[1]. The energy barrier for absorption of a Si atom in the first layer was found to be 0.617 eV from ab-initio calculations[4], while experiments found 0.43 eV[2] and we came up with a value of 0.50 eV, all these values being of the same order of magnitude, even if the ab-initio calculations are significantly higher than the experimental results. As regards the energy barrier for diffusion of a Si adatom on the Ag(111) substrate, different values can be inferred from the literature. Ab-initio calculations lead to different energy barriers: Shu et al. found two successive barriers between the hcp and fcc sites of 0.14 then 0.16 eV[5], while Satta and co-workers found a single asymmetric barrier of 0.124 eV in one direction and 0.058 eV in the other[4], and finally Gao and Zhao calculated a barrier of 0.031 eV[6]. Based on a simplified model of irreversible aggregation that minimizes barriers, Prevo1t and co-workers deduced a value of 0.26 eV from the experiments[2]. In our analysis, we find an energy barrier for the diffusion of Si on Ag(111) of $3 \times E_{Ag}^{Si,out}(h=1) = 0.30$ eV, that matches experiments at low temperature (when intermixing is not important). The discrepancy between values deduced from experiment and statistical analysis on the one hand, and ab-initio calculations that indicate much lower values on ideal systems on the other, may be related to additional surface effects (diffusion pathways, surface stress, etc)[3,7,8] and merits further investigation. Moreover, we note that the energy barriers $E_{inter}^{abs}(h \leq 0)$ and $E_{inter}^{des}(h \leq 0)$ for penetration of Si atoms into the deep layers of the substrate, that are estimated at 1 eV, are essentially large enough for deep-layer intermixing to be inhibited in the simulations. It should be regarded as a lower value for consistency with experiments. The value of 2.567 eV calculated by Satta and coworkers[4] is indeed larger, and therefore compatible with experiments. Finally, if energy barriers are tricky, barrier differences can be related to the energies of stable positions. Ab-initio calculations can thus link the difference between the two barriers $E_{inter}^{des} - E_{inter}^{abs}$ with the result 0.11 eV in the GGA approximation and -0.08 eV in the LDA approximation,[2]. Our estimate of 0.10 eV in Table 1 compares favorably with the GGA approximation.

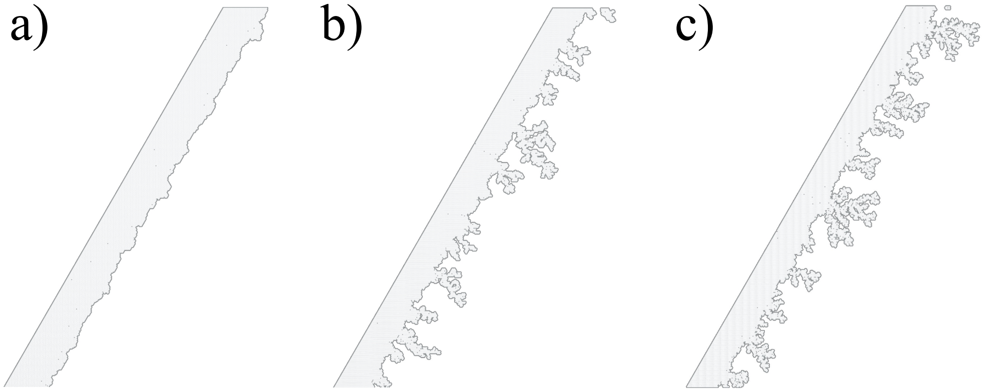

Figure 1. Simulations of the deposition of 0.1 ML of Ag(111) on a vicinal surface (step atoms in grey) at room temperature, with the experimental flux $F = 0.1$ ML/h, for $E_{Ag}^{Ag,in}(h=1) = 0.30$ eV (a), 0.35 eV (b) and 0.40 eV (c). Surface effects are neglected and energies are assumed not to depend on h. The simulated region is a rhombus with an angle of 60 degrees and consisting of 400 atoms on each side, so that the length of each side is almost 115 nm on Ag(111).

References

1. Brune, H. *et al.* Effect of strain on surface diffusion and nucleation. *Phys. Rev. B* **52**, R14380, DOI: 10.1103/PhysRevB.52.R14380 (1995).

2. Bernard, R., Borensztein, Y., Cruguel, H., Lazzeri, M. & Prévot, G. Growth mechanism of silicene on Ag(111) determined by scanning tunneling microscopy measurements and ab initio calculations. *Phys. Rev. B* **92**, 045415, DOI: 10.1103/PhysRevB.92.045415 (2015).

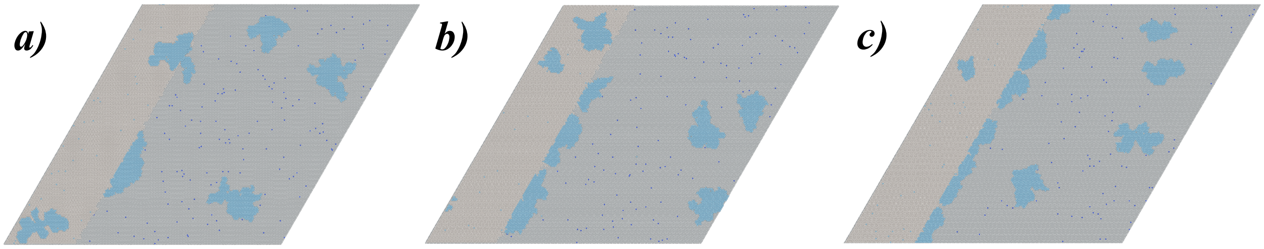

Figure 2. Simulations of the deposition of 0.1 ML of Si on a Ag(111) surface (step atoms in grey) at $T = 200$ K, with the experimental flux $F = 0.1$ ML/h, for $E_{Si}^{Ag,in} = 0.02$ eV (a), 0.03 eV (b) and 0.04 eV (c). For small values of $E_{Si}^{Ag,in}$, the step-edge is poorly decorated with Si, while it is continuously decorated for intermediate values, and intermixing occurs in the islands for higher values. Surface effects are neglected and energies are assumed not to depend on h. The simulated region is a rhombus with an angle of 60 degrees and consisting of 400 atoms on each side ($\sim$ 115 nm).

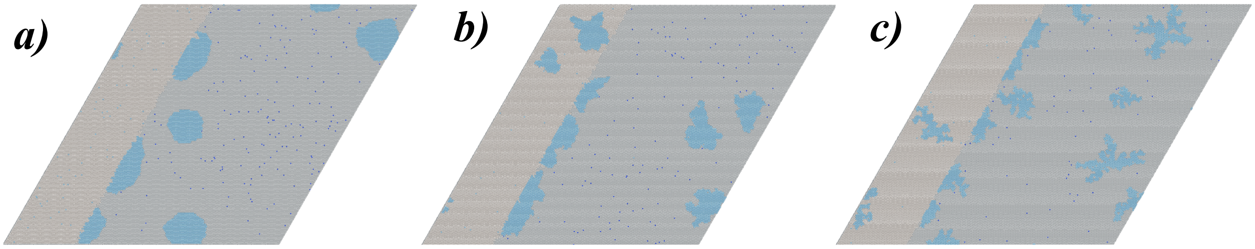

Figure 3. Simulations of the deposition of 0.3 ML of Si on a Ag(111) surface at $T = 200$ K, with the experimental flux $F = 0.1$ ML/h, for $E_{Si}^{Si,in} = 0.08$ eV (a), 0.10 eV (b) and 0.12 eV (c). The islands shapes are rather compact and facetted for low values of $E_{Si}^{Si,in}$, rather compact but irregular at intermediate values, and dendritic at large values. Surface effects are neglected and energies are assumed not to depend on h. The simulated region is a rhombus with an angle of 60 degrees and consisting of 400 atoms on each side ($\sim$ 115 nm).

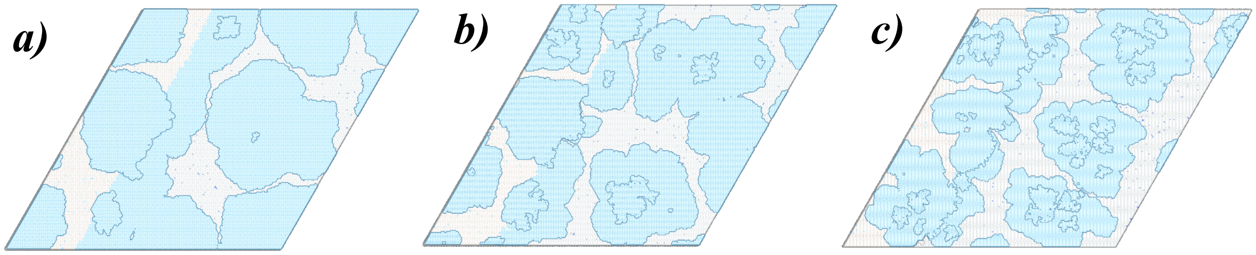

Figure 4. Simulations of the deposition of 0.8 ML of Si on a Ag(111) surface at $T = 200$ K, with the experimental flux $F = 0.1$ ML/h, for $E_{Si}^{Si,out} = 0.11$ eV (a), 0.12 eV (b) and 0.13 eV (c). Surface effects are neglected and energies are assumed not to depend on h. The simulated region is a rhombus with an angle of 60 degrees and consisting of 400 atoms on each side ($\sim$ 115 nm).

3. Ratsch, C., Seitsonen, A. P. & Scheffler, M. Strain dependence of surface diffusion: Ag on ag(111) and pt(111). *Phys. Rev. B* **55**, 6750, DOI: 10.1103/PhysRevB.55.6750 (1997).

4. Satta, M., Colonna, S., Flammini, R., Cricenti, A. & Ronci, F. Silicon reactivity at the ag(111) surface. *Phys. Rev. Lett.* **115**, 026102, DOI: 10.1103/PhysRevLett.115.026102 (2015).

5. Shu, H. *et al.* Two-dimensional silicene nucleation on a ag(111) surface: structural evolution and the role of surface diffusion. *Phys. Chem. Chem. Phys.* **16**, 304, DOI: 10.1039/C3CP53933D (2014).

6. Gao, J. & Zhao, J. Initial geometries, interaction mechanism and high stability of silicene on ag(111) surface. *Sci. Rep.* **2**, 861, DOI: 10.1038/srep00861 (2012).

7. Xian, L. & Chou, M. Y. Diffusion of si and c atoms on and between graphene layers. *J. Phys. D: Appl. Phys.* **45**, 455309, DOI: 10.1088/0022-3727/45/45/455309 (2012).

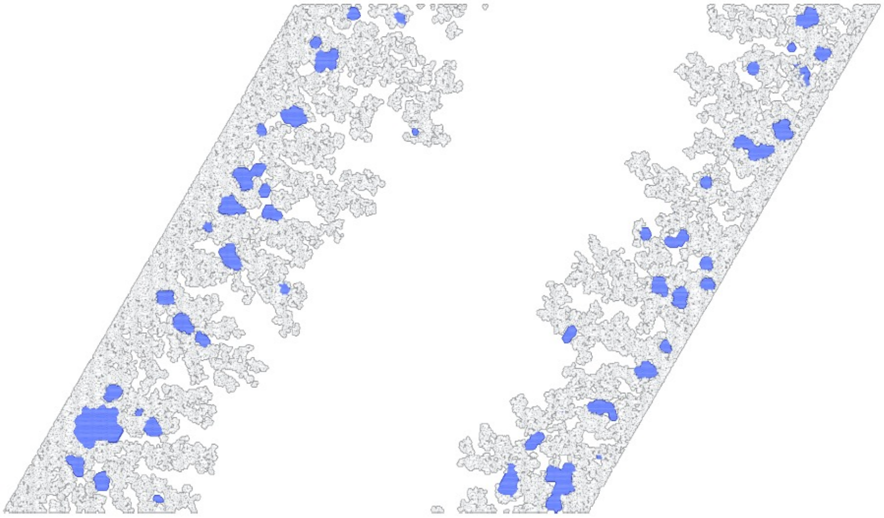

Figure 5. Simulations of the deposition of 0.3 ML of Si on a Ag(111) surface when surface effects are neglected so that energies are assumed not to depend on h. The growth temperature is $T = 300\,\mathrm{K}$, and the deposition flux is the experimental value $F = 0.1$ ML/h, with the first trial parameters. The simulated region is a rhombus with an angle of 60 degrees and consisting of 400 atoms on each side ($\sim 115\,\mathrm{nm}$).

8. Liu, K. *et al.* Capillary-driven elastic attraction between quantum dots. *Nanoscale* **11**, 7798 (2019).
